# Robot-Assisted Autonomous Reduction of a Displaced Pelvic Fracture: A Case Report and Brief Literature Review

**DOI:** 10.3390/jcm11061598

**Published:** 2022-03-14

**Authors:** Yufeng Ge, Chunpeng Zhao, Yu Wang, Xinbao Wu

**Affiliations:** 1 Department of Trauma and Orthopaedics, Peking University Fourth School of Clinical Medicine, Beijing Jishuitan Hospital, Beijing 100035, China; geyufeng_jst@163.com (Y.G.); zcpllza2008@163.com (C.Z.); 2 Key Laboratory of Biomechanics and Mechanobiology, Beihang University, Ministry of Education, Beijing 100083, China; wangyu@buaa.edu.cn; 3 Beijing Advanced Innovation Center for Biomedical Engineering, School of Biological Science and Medical Engineering, Beihang University, Beijing 100083, China

**Keywords:** case report, robot-assisted surgery, closed reduction, pelvic fracture

## Abstract

Displaced pelvic fracture is among the most complicated fractures in traumatic orthopedics, with high mortality and morbidity. Reduction is considered a complex procedure as well as a key part in surgical treatment. However, few robotic techniques have been employed in the reduction of pelvic fracture, despite the rapid advancement of technologies. Recently, we designed a robot surgery system specialized in the autonomous reduction of displaced pelvic fracture and applied it in the true patient for the first time. In this paper, we report its successful clinical debut in the surgery of a displaced pelvic fracture. Total surgery time was 110 min and an anatomic reduction was achieved. We then present a brief overview of the literature about reduction techniques in pelvic fracture and introduce related principles involved in our robot-assisted reduction system.

## 1. Introduction

Surgery of displaced pelvic fractures (PF) has always been challenging for orthopedic surgeons. It requires expertise in 3D pelvic anatomy, available reduction techniques, and fixation strategies in most cases. To achieve a satisfactory reduction, many surgeons have gone to great lengths to optimize differing approaches [1,2,3]. However, it is essential to emphasize that the holy grail of “steps and technique of reduction” remains an art more than a science, and the extensive open surgery was reportedly associated with a high risk of neurovascular injury leading to poor prognosis. With the rapid development of technology in the 21st century, robot techniques have become a powerful tool and an emerging art assisting surgeons to eliminate all kinds of complex barricades [4,5]. Some have been prevailing in PF fixation [6,7,8], but few have been introduced to handle the issue of PF reduction, which is both a highly skilled and physically demanding procedure, and often time-consuming.

**Figure 1:**
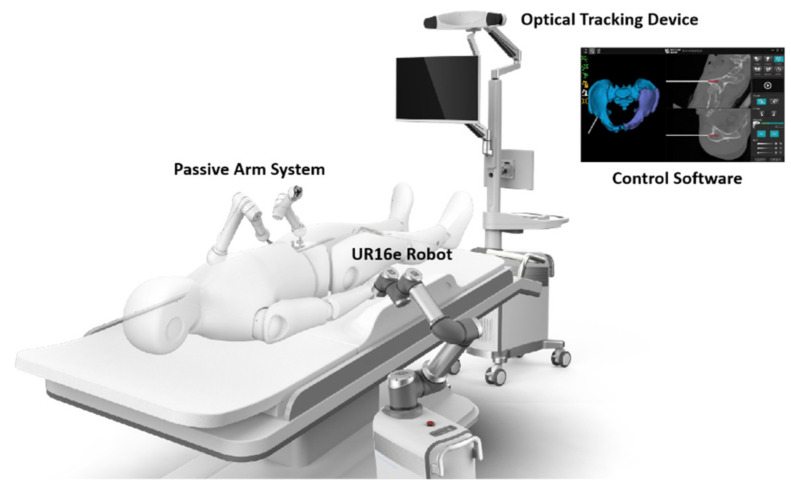
Diagram of the robot system.

We recently designed a new robot system that could autonomously identify the pelvic fracture pattern and plan and execute the reduction approach. Our robot system consists of one central part and its assistance part. The central part has three components (Figure 1): an optical tracking device, a control software including planning and navigation algorithm, which was described in our previous study [9], and a robotic arm system. The assistance part includes two components. One is an elastic traction system, which was described before in another study to facilitate fracture reduction and help reduce the force applied by robotic arm [10]. The other is a passive arm system, which has two 9-degree of freedom (DOF) passive holders installed at the healthy hemipelvis side and helps firmly fix the pelvis. In the central part, the optical tracking device (NDI Polaris Vega) employs an infrared stereo camera and reference frames to acquire intraoperative 3D images and locate every operation on the pelvis. After that, the planning and navigation software [9] mirrors the healthy hemipelvis and develops a trajectory to reduce the fracture. Then, the 6-DOF robotic manipulator (UR16e) built on a mobile platform completes the reduction work under the guidance of the control software and optical tracking device by holding three Schanz screws.

In this report, we present the first case of robot-assisted autonomous reduction for displaced PF followed by percutaneous screw fixation and review related literature in this area. We believe this technique has enormous potential for PF treatment and merits further studies.

## 2. Case Report

A 56-year-old male presented to the accident and emergency department six hours after hit by a heavy object directly on the left hip. He was unable to walk after the injury. On arrival, the patient was hemodynamically stable with a Glasgow Coma Score of 15/15.

**Figure 2:**
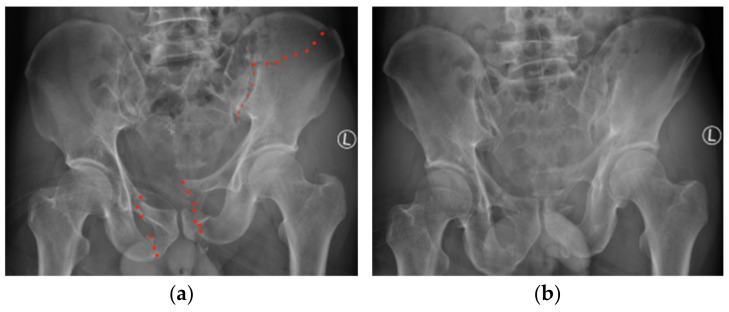

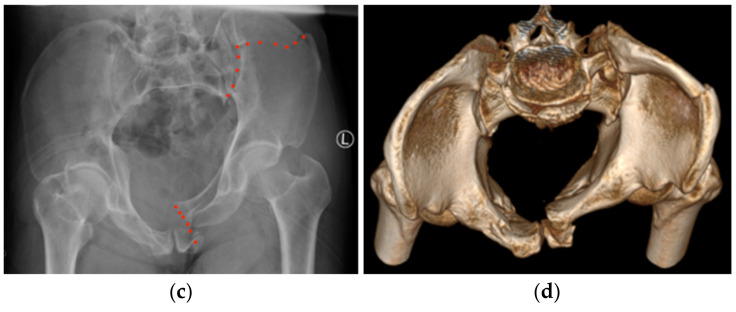
Imaging of the displaced pelvic fracture with fracture mapped by red dotted line: (**a**) AP view; (**b**) outlet view; (**c**) inlet view; (**d**) 3D reconstruction view.

On physical examination, the patient had tenderness on palpation of the right sacroiliac joint and symphysis pubis. No apparent limb length discrepancy was noted, and distal neurovascular structures were intact. Digital rectal examination and focused assessment with sonography in trauma scans were normal. Pelvic compression and distraction tests were not performed due to the pain. Radiographs of pelvic series (AP, inlet, outlet views) (Figure 2a–c) and computed tomography (CT) scan with 3D reconstruction (Figure 2d) showed an unstable pelvic injury (Tile B2), with a fracture line extending from the iliac crest to the sacroiliac (SI) joint posteriorly (crescent-shaped pelvic fracture) and rupture of superior and inferior rami anteriorly on both sides. A marked internal rotation of the fragment existed.

**Figure 3:**
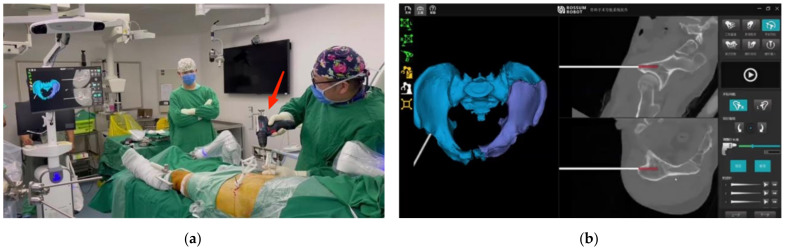
Gripping screws placement under navigation: (**a**) picture taken during gripping screws placement; (**b**) real time monitoring.

After the approval by the Ethics Committee and informed consent, a surgery of robot-assisted closed reduction and percutaneous internal fixation for pelvic fracture was planned three days after injury. A flowchart was made to outline our surgery procedure (Figure S1 in the Supplementary Materials). With the patient supine, his pelvis and the leg of the affected side (left side in our present case) were prepped and draped free. According to preoperation planning, the first step of our surgery was to achieve full preparation (such as setup, traction, etc.) for the next reduction and fixation. Two SI guidewires were temporarily placed on the healthy side (right side in our case) with the help of a tracker-drill, which made the final SI screw fixation much easier. Then, trackers were assembled on both sides, followed by cone beam computed tomography (CBCT) scan to obtain the image registration. Next, distal femoral traction utilizing the elastic traction device was applied on the left side to facilitate the subsequent reduction. Several gripping screws (3 left side, 2 right side, 5.0 mm size, 40 mm threaded, 200 mm length) were placed on the ilium for the arms to hold firmly (Figure 3), and both the passive (right) arms and the robotic (left) arm were prepped and connected to the corresponding gripping screws.

**Figure 4:**
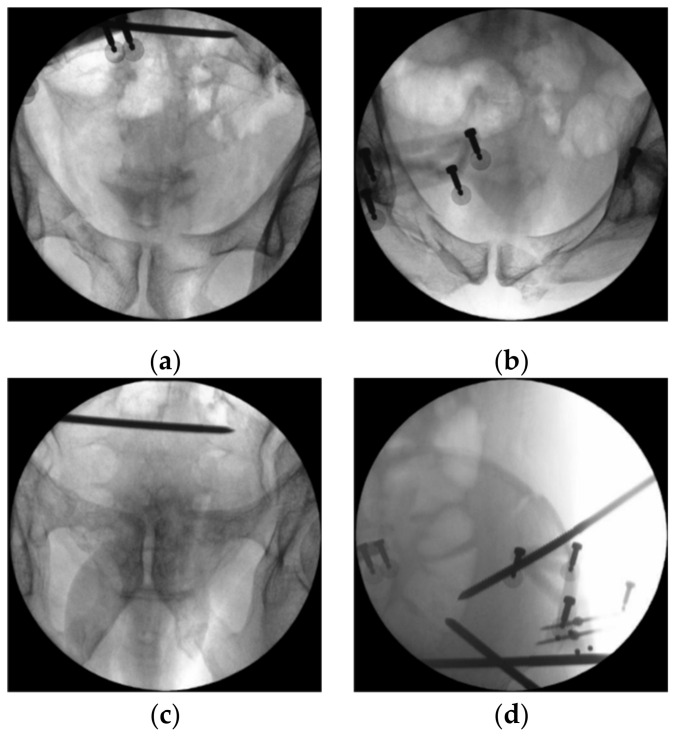
Intraoperative fluoroscopy after robot-assisted reduction: (**a**) AP view of anterior ring; (**b**) inlet view of anterior ring; (**c**) outlet view of anterior ring; (**d**) fracture site at the iliac crest.

The second step was to reduce the displaced fracture based on pelvis symmetry. The software used at this step was proved effective in our model study before [9]. According to the template reconstructed from mirroring the relatively intact hemipelvis, the software autonomously programmed a reduction trajectory to move the fractured fragment to the presumptive site (Video S1 in the Supplementary Materials). That pelvic morphology was structured using the mirror of more than ninety thousand points at the healthy side (right side) [11]. Following the completed reduction, in our case, intraoperative fluoroscopy was used to examine the reduction quality (Figure 4). All the views illustrated a satisfactory opposition between the fragments. Furthermore, a generally available software, Geomagic Studio 2013 (Geomagic Inc., Chino, CA, USA), was used to measure the global 3D point cloud error for each set of registration result [9]. The average error was 1.18 mm, and 99.97% points revealed an error less than 4 mm.

After validation, the third step was the definitive percutaneous fixation. In our present case, the left SI joint was then stabilized with two 6.5 mm size, 100 mm length cannulated partially threaded cancellous screws. Another two percutaneous lag screws were placed retrogradely with the help of navigation. Next, a stress test was conducted under the image intensifier to confirm the rotational stability of the fixed pelvis. No intraoperative complication was noted. The total surgery time was 110 min, and blood loss 50 mL.

**Figure 5:**
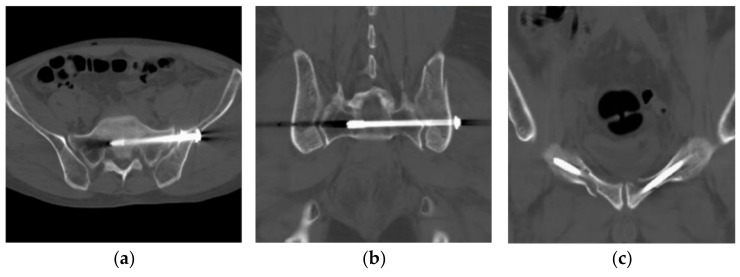
Post-operative CT scan to identify the reduction quality: (**a**) axial plane at the SI joint; (**b**) coronal plane at the SI joint view; (**c**) coronal plane at the anterior fracture site.

The post-operative period was uneventful. A CT scan on the day after surgery showed a maximal displacement of 2.8 mm based on multiple plane reconstruction (Figure 5), which is classified as excellent reduction quality according to Matta criteria [2] (excellent at 4 mm or less, good at 5–10 mm, fair at 10–20 mm, and poor at more than 20 mm). The patient started the active and passive joint movements and strength exercises on the bed from the second post-operative day. We advised non-weight bearing or toe-touch weight-bearing for 2 weeks, and then progressive weight could be added depending on pain. At the 3-month follow-up outpatient appointment, the patient could walk without a cane and had no limitation of daily living. Function assessment showed a Majeed score [12] of 95/100, categorized as an excellent outcome. The radiographs revealed no problem (secondary displacement, non-union, hardware failure).

## 3. Discussion

Due to the irregular shape and complexity of neurovascular structure around the pelvis, reduction and fixation of a displaced pelvic fracture remains complicated for most surgeons. Since the last century, various orthopedic instruments and techniques have been introduced to tackle such tough fracture [1,2,13,14,15,16,17]. In the late 1990s, some pioneers [2,13,16,17], such as Tile, Matta, Routt, Kellam, to name but a few, elaborated their experience of open reduction and internal fixation (ORIF) for displaced PF. Since then, the approaches they described have, to a certain extent, become a classic for every orthopedic surgeon to learn. However, several significant issues were encountered as we conducted such methods for decades. First, the surgery is full of challenges and often associated with an increased risk of neurovascular injury. Therefore, the learning curve for an inexperienced surgeon is long. Second, such extensive open surgery is unsuitable and often seen as a second hit for a polytrauma patient. Sometimes, patients may have to endure delayed treatment due to the complexity of their specific fractures. Third, we also want to mention that it remains hard to achieve anatomic reduction due to its morphological features, despite reducing through open surgery. For example, while reducing the posterior ring, it may seem that alignment is achieved on the anterior surface of the SI joint via touching and fluoroscopy. However, due to indiscoverable rotations, a posterior gap or disagreement could probably occur.

Considering the drawbacks of open surgery, more and more pathfinders have been looking for a minimally invasive way to handle pelvic fracture, and closed reduction followed by percutaneous screw fixation has become a growing trend [14,15,17,18]. Placements of Schantz pins in the iliac crest, antero-superior iliac spine and supra-acetabulum are used to reduce the antero-posterior displacement and rotational deformity while axial traction to the lower limb for the superior displacement [17]. Matta [15] then introduced a specialized orthopedic system for PF reduction to facilitate the healthy side fixed to the table. Lefaivre [14] later designed a Starr frame to reduce displaced PF, stabilizing the intact hemipelvis and freeing surgeons’ hands once the fracture reduced. However, the reduction process was still conducted by surgeons and was too dependent on surgeons’ personal experience. Inspired by Lefaivre, Zhao et al. [18,19] then developed a computer-aided reduction mechanism and combined it with the Starr frame. An intraoperative 3D reconstruction model was applied to measure the reduction quality, and computer software was used to calculate the residual differences between the actual and virtual anatomical reduction positions. Still, the reduction accuracy relied on the surgeons, and after each movement, an intraoperative CT scan or fluoroscopy was required to check the result. Overdose radiation and reduction quality were two aspects impeding its development.

With the advancement of orthopedic surgery robots and computer-assisted surgery techniques, real-time operation under navigation has facilitated the reduction and fixation of displaced PF to a great extent [20,21]. To overcome the difficulties such as radiation exposure and reduction quality in PF, our department designed the new surgical robot employing several primary techniques, described herein (co-designed by Beijing Jishuitan Hospital and Rossum Robot—Beijing, China). First, the autonomous reduction planning adopts the principle of mirroring and utilizes the symmetry of the pelvis, which we called Pelvic Symmetry Reduction (PSR), introduced in our previous study [11]. The pelvis symmetry has been well established and verified. In Ead’s study [22,23], she demonstrated the high degree of pelvis symmetry and attempted to reconstruct a fractured pelvis through her mirroring tools. Wang et al. [24] also confirmed the symmetry and then found the virtual plane on which the intact healthy hemipelvis could mirror. Our team also explored the symmetry beforehand, using the PSR method to simulate reduction on fractured pelvis models, and indicated its potential feasibility in developing a surgery robot [11]. Additionally, now, the newborn robot made its clinical debut.

The second primary technique is the elastic traction device. In order to overcome the very significant forces exerted by the muscles and ligaments during reduction, lots of effort, such as axial traction of the lower limb, is required. Traditionally, manual traction by surgeons or rigid traction using the conventional traction table is employed to ease the reduction. However, the former wastes the workforce, while the latter greatly limits the flexibility of the reduction process. In order to minimize the load on the robotic arm and solve the difficulties mentioned above, we designed an elastic traction device which was described in detail before [10], and listed it as a necessary component of our robot system. After reduction, the surgeon performed the final fixations manually while the robot arm firmly maintained the reduction. The total surgery time was 110 min and blood loss 50 mL. Only two surgeons are involved, other than one scrub nurse and one technician. It is recognized that the time and blood loss was tightly associated with the fracture pattern and surgical approach and was based largely on each individual patient. Ma [25] retrospectively studied 128 patients with pelvic fracture and reported a median surgery time of 103 min in minimally invasive surgery (MIS) and 152 min in ORIF surgery. He also concluded the estimated blood loss showing an average 50 mL in MIS and 250 mL in ORIF. Our results were similar to that of MIS, which revealed evident advantage over extensive open surgery. In Ma’s study, MIS group received 77.4% (48/62) excellent reduction, while the ORIF group saw 75.8% (50/66). Five patients (7.5%) in ORIF group yielded perioperative complications (surgical site infection, unplanned re-operation, malunion). In our presented case, an anatomic reduction was obtained and no complication occurred. We believe the robot-assisted reduction system will have tremendous potential to benefit both patients and surgeons in future PF management, although more cases and more studies are required in the future.

To the best of our knowledge, this is the first case report on robot-assisted surgery for pelvic fracture. Such a technique is less demanding with respect to both the skills and staff involved, and saves both time and labor. Theoretically, there is no radiation exposure, and the reduction accuracy could be monitored through point cloud calculation. In our presented case, we used intraoperative fluoroscopy only to verify our reduction results. However, there are still some disadvantages we may overlook, such as the squeezing of surrounding vessels and nerves, which were not visible in our case. In addition, the indications of this robot-assisted reduction method have not been made clear. Future studies should be planned to optimize our surgical robotic system.

This case report also has some limitations. First, only one case was performed. More cases are required before concluding the reliability of this new robot. Second, comparative studies are further needed to investigate its superiority over other approaches.

## Data Availability

Data sharing not applicable.

## Supplementary Materials

The following supporting information can be downloaded at: https://www.mdpi.com/article/10.3390/jcm11061598/s1, Figure S1: Flowchart of surgery procedure; Figure S2: Post-operative X-ray (AP, outlet, inlet views); Video S1: Autonomous reduction procedure.
