# Supplementary figures and images for: Robot-Assisted Autonomous Reduction of a Displaced Pelvic Fracture: A Case Report and Brief Literature Review
Source: J Clin Med. 2022 Mar 14;11(6):1598. doi: 10.3390/jcm11061598 (PMC8950953; doi:10.3390/jcm11061598)

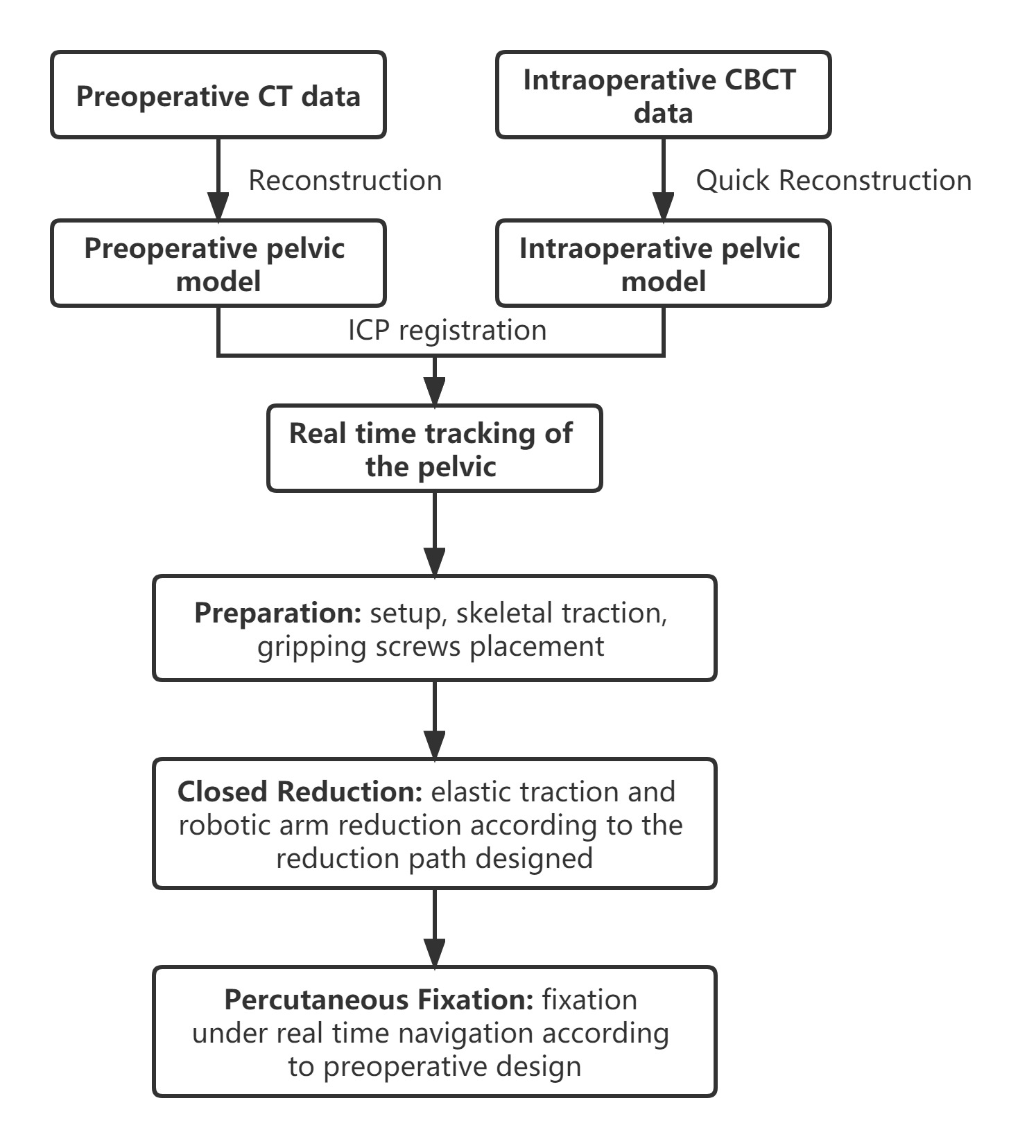

Supplement: Supplementary file 1 [file jcm-11-01598-s001.zip › Figure S1. Flowchart of surgery procedure.jpg]

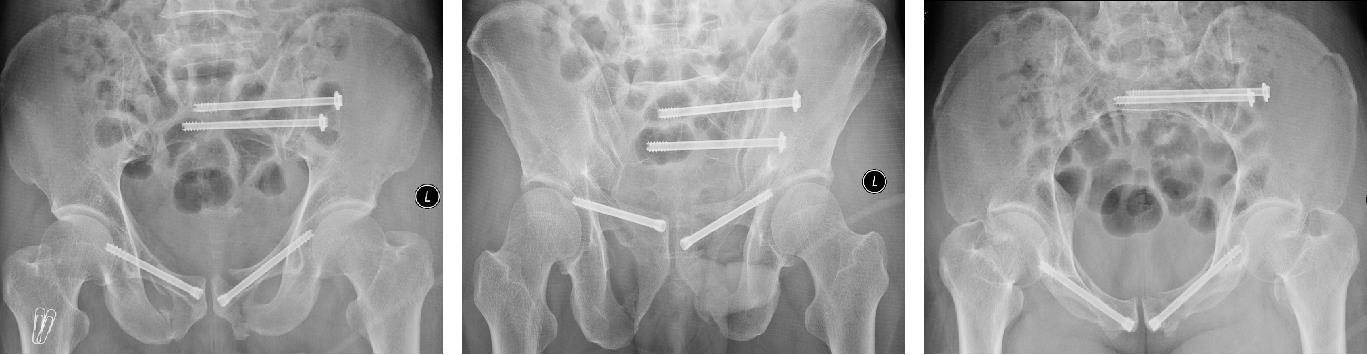

Supplement: Supplementary file 1 [file jcm-11-01598-s001.zip › Figure S2. Post-operative X-ray (AP, outlet, inlet views).jpg]
